# Oxidative stress and dysregulated long noncoding RNAs in the pathogenesis of Parkinson’s disease

**DOI:** 10.1186/s40659-025-00585-7

**Published:** 2025-01-27

**Authors:** Jialu Wang, Meitong Liu, Jiuhan Zhao, Pan Hu, Lianbo Gao, Shen Tian, Jin Zhang, Huayan Liu, Xiaoxue Xu, Zhenwei He

**Affiliations:** 1 Department of Neurology, First Affiliated Hospital of China Medical University, No.155 Nanjing North Street, Heping District, Shenyang, 110001 Liaoning China; 2 Department of Neurology, Fourth Affiliated Hospital of China Medical University, No.4 Chongshan East Road, Huanggu District, Shenyang, 110032 Liaoning China; 3 Key Laboratory of Neurological Disease Big Data of Liaoning Province, No.155 Nanjing North Street, Heping District, Shenyang, 110001 Liaoning China

**Keywords:** Long-noncoding RNAs, Oxidative stress, Reactive oxygen species, Neurodegeneration, Parkinson’s disease

## Abstract

Parkinson’s disease (PD) is a progressive age-related neurodegenerative disease whose annual incidence is increasing as populations continue to age. Although its pathogenesis has not been fully elucidated, oxidative stress has been shown to play an important role in promoting the occurrence and development of the disease. Long noncoding RNAs (lncRNAs), which are more than 200 nucleotides in length, are also involved in the pathogenesis of PD at the transcriptional level via epigenetic regulation, or at the post-transcriptional level by participating in physiological processes, including aggregation of the α-synuclein, mitochondrial dysfunction, oxidative stress, calcium stabilization, and neuroinflammation. LncRNAs and oxidative stress are correlated during neurodegenerative processes: oxidative stress affects the expression of multiple lncRNAs, while lncRNAs regulate many genes involved in oxidative stress responses. Oxidative stress and lncRNAs also affect other processes associated with neurodegeneration, including mitochondrial dysfunction and increased neuroinflammation that lead to neuronal death. Therefore, modulating the levels of specific lncRNAs may alleviate pathological oxidative damage and have neuroprotective effects. This review discusses the general mechanisms of oxidative stress, pathological mechanism underlying the role of oxidative stress in the pathogenesis of PD, and teases out the mechanisms through which lncRNAs regulate oxidative stress during PD pathogenesis, as well as identifies the possible neuroprotective mechanisms of lncRNAs. Reviewing published studies will help us further understand the mechanisms underlying the role of lncRNAs in the oxidative stress process in PD and to identify potential therapeutic strategies for PD.

## Background

Parkinson’s disease (PD), also known as paralysis tremor, is a neurodegenerative disease characterized by persistent loss of dopaminergic neurons in the substantia nigra dense region [1]. PD affects approximately ten million people globally and has a prevalence of approximately 0.3% in developed countries [2]. In China, the prevalence of PD in people over 60 years of age is approximately 1.0% [3]. The number of patients with PD is increasing annually as the global population ages, greatly affecting the quality of life of patients and their families [4]. The main pathological feature associated with PD is the gradual loss of dopaminergic neurons in the substantia nigra of the brain that leads to reduced dopamine (DA) in the striatum, resulting in pathophysiological changes in the downstream basal ganglia circuit and subsequent motor dysfunction [5]. Motor symptoms of PD include myotonic, bradykinesia, static tremor, and frozen gait [6]. Non-motor symptoms may accompany motor symptoms and include depression, memory disorders, and autonomic dysfunction [7]. Few drugs are currently effective against PD. The first line treatment is levodopa. However, its clinical use is limited due to its multiple adverse reactions and insensitivity in some patients [8]. Thus, studying the pathogenesis of PD and exploring new therapeutic strategies for the disease is extremely urgent. Oxidative stress plays a key role in the pathogenesis of PD, and together with neuroinflammation, mitochondrial dysfunction, and dopamine metabolism, can result in oxidative cell damage, leading to degeneration and necrosis of DA neurons [9–11].

Oxidative stress can be considered as an imbalance between oxidation and anti-oxidation in the body [12]. The body is more inclined to oxidation and produces a large number of reactive oxygen species (ROS) [13]. ROS are oxygen-containing molecules with high biological activity. They include superoxide anions, hydrogen peroxide, and hydroxyl radicals, which are generated by exogenous oxidants or intracellular aerobic metabolism [14]. ROS have important physiological functions, prevent the invasion of foreign substances, and act as regulators of internal biological processes [15]. However, when the levels of ROS exceed the physiological requirements of cells, they can destroy the integrity of the cell structure and cell function through oxidative degradation of key molecules such as DNA, proteins, and lipids [16]. Recent studies have suggested that oxidative stress is associated with the occurrence and development of PD [17, 18].

Long noncoding RNAs (LncRNAs) are noncoding RNAs with lengths greater than 200 nucleotides that are located in the nucleus and cytoplasm [19]. Although they do not encode proteins themselves, they can regulate various cellular biological processes, including cell transcription, histone modification, and DNA methylation [20]. Therefore, lncRNAs are new potential biomarkers of biological function [21]. LncRNAs are highly expressed in the central nervous system (CNS) and can be detected using high-throughput techniques such as in situ hybridization, microarray analysis, and RNA sequencing [22]. Some lncRNAs are differentially expressed in PD, resulting in protein misfolding and aggregation, mitochondrial dysfunction, oxidative stress, autophagy, apoptosis, and neuroinflammation, leading to the pathogenesis associated with PD [23, 24].

LncRNAs are generally studied separately from oxidative stress. However, the two are inextricably intertwined during neurodegeneration. LncRNAs regulate many oxidative stress response genes and pathways, while oxidative stress affects the expression of various lncRNAs [25, 26]. LncRNAs and oxidative stress also affect other processes associated with neurodegeneration, including mitochondrial dysfunction and increased neuroinflammation that ultimately lead to neuronal death [27, 28]. Reviewing the mechanism through which lncRNAs regulate oxidative stress will give us a deeper understanding of the specific roles of the two components in neurodegeneration and may provide new perspectives for neuroprotection in PD.

## Regulatory mechanisms of LncRNAs

LncRNAs generally refer to noncoding RNA transcripts that are greater than 200 nucleotides in length [29]. Noncoding sequences were discovered in the 1970s, and were initially called “junk DNA” based on prevailing evolutionary theories that predicted no biological function [30]. As research has progressed, studies have shown that what was once “junk DNA” may actually have multiple important biological functions [31]. Recent studies have shown that lncRNAs play important roles in regulating normal cell development and function [32]. In addition, the role of lncRNAs in neurodevelopment, regeneration, and neurodegenerative diseases has become a research hotspot [33]. A growing body of evidence has illustrated the critical role of noncoding RNAs (ncRNAs) in multiple biological processes and in various neurodegenerative diseases such as Alzheimer’s disease, PD, and Huntington’s disease [34–36].

LncRNAs have regulatory roles at multiple levels, and the development and differentiation process of the CNS also requires the participation of lncRNAs. LncRNAs can be divided into five categories based on the relative positions of lncRNA sequences and protein-coding genes: (1) Sense lncRNAs: lncRNA sequences overlapped with protein-coding genes; (2) Antisense lncRNAs: lncRNA sequences overlapped with antisense chains of protein-coding genes; (3) Bidirectional lncRNAs: lncRNA sequences transcribed from different bidirectional promoters relative to protein-coding genes; (4) Intronic lncRNAs: The complete lncRNA sequence is derived from the transcriptional intron and can be an independent transcript or precursor mRNA processing product; (5) Intergenic lncRNAs: lncRNA sequences are located between protein-coding genes but do not overlap [37, 38].

LncRNAs play a key role in development and differentiation. Deep sequencing of tissues and cells showed that, as regulators of gene expression, the functions of lncRNAs can be divided into three based on their regulatory methods: (1) Transcriptional regulation: lncRNAs can induce chromatin remodeling and modification, and serve as scaffolds or bridges for proteins or chromatin [39]. (2) Post-transcriptional regulation: lncRNAs can bind to mRNAs through base complementary pairs to block the splicing sites of spliceosomes, resulting in spliced transcripts, mRNA denaturation, translation inhibition, or the generation of endogenous small interfering RNA [40]. (3) Interactions with other biomolecules: lncRNAs can bind to specific protein chaperones to regulate protein activity, act as decoys to change protein localization, act as scaffolds to allow the formation of larger RNA-protein complexes, or act as miRNA sponges to interact with miRNAs [41].

## Mechanism underlying oxidative stress in PD

Under normal physiological conditions, oxidation and antioxidant functions in the body are at equilibrium [42]. When the body is affected by external influences, its antioxidant capacity decreases, and free radicals cannot be removed in a timely manner, resulting in the accumulation of free radicals that disrupt this equilibrium, promoting the development of PD [17, 43].

ROS include superoxide anions, hydrogen peroxide, and hydroxyl radicals, which are by-products of mitochondrial metabolism [44]. Superoxide produced by the mitochondria can be reduced to H_2_O_2_, which is broken down into oxygen and water by catalase [45]. However, under pathological conditions, H_2_O_2_ is released into the cytoplasm due to damage to the catalase, resulting in oxidative stress [46]. H_2_O_2_ can also generate hydroxyl radicals via Fenton reaction under the action of reducing metals such as iron [47]. Free hydroxyl radicals are one of the most harmful free radicals of all ROS [48]. Once produced, they can immediately react with surrounding substances, resulting in oxidative stress [49]. The main sites of ROS production in cells include mitochondrial electron transport chain (ETC), endoplasmic reticulum, and reduced nicotinamide adenine dinucleotide phosphate oxidase (NOX) complex [50]. Of these, mitochondrial electron transfer is considered the main source of ROS in the body [51].

**Fig. 1:**
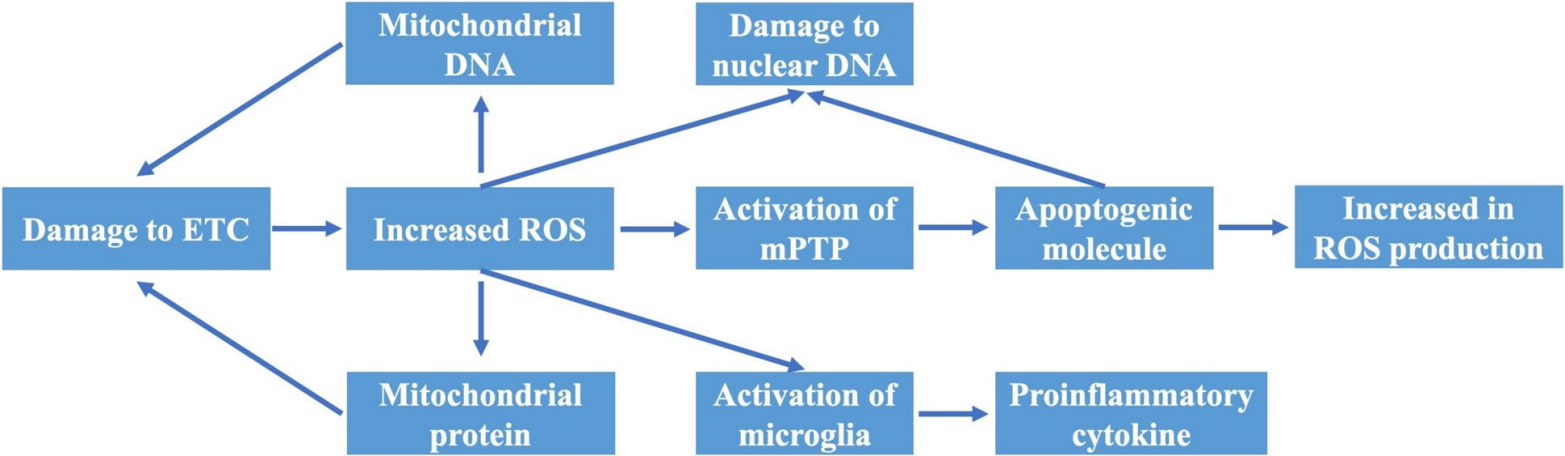
ROS trigger a cascade of events that lead to the degeneration of neurons. Oxidative stress plays a central role in neurodegeneration, creating a vicious cycle by triggering cascading events, including mitochondrial dysfunction, nuclear and mitochondrial DNA damage, and neuroinflammation, which lead to the production of more ROS [16]. ETC, electron transport chain; mPTP, mitochondrial permeability transition pore; ROS, reactive oxygen species

Mitochondria, as one of the main sites of ROS production, are particularly vulnerable to oxidative stress damage [52]. Unlike nuclear DNA, mitochondrial DNA (mtDNA) is not protected by histones and, as a result, is vulnerable to oxidative damage [53]. Since most proteins encoded by mtDNA are involved in the ETC, mutations and deletions in mtDNA may interfere with the ETC and increase ROS formation, resulting in a cycle that leads to further mitochondrial damage. The ETC is the mitochondrial system responsible for energy production. Damage to the ETC leads to mitochondrial dysfunction, which in turn affects cellular energy metabolism. Impairment of the ETC may result in abnormal functioning of mitochondrial proteins, exacerbating the mitochondrial dysfunction. Mitochondrial dysfunction is associated with increased production of ROS, which are highly reactive chemicals capable of damaging cell membranes, proteins, and DNA. Elevated ROS levels initiate oxidative stress, a condition of cellular damage that can lead to cell death and inflammation [54]. Oxidative stress and increased ROS can activate microglia, the immune cells of the brain, which play a significant role in the inflammatory response. Activated microglia release a array of pro-inflammatory cytokines, including tumor necrosis factor-α (TNF-α), interleukin-1β (IL-1β), and interleukin-6 (IL-6), which can intensify inflammation and neuronal damage [55] (Fig. 1). Moreover, in DA neurons, the active form of matrix metalloproteinase 3 (MPP-3) increases with oxidative stress, and MPP-3 activates microglia to produce ROS [56]. Therefore, alternative strategies are necessary for neutralizing the toxic effects of ROS and restoring the redox balance in neurodegenerative cells during PD therapy [57].

## Nrf2-ARE signaling pathway

**Fig. 2:**
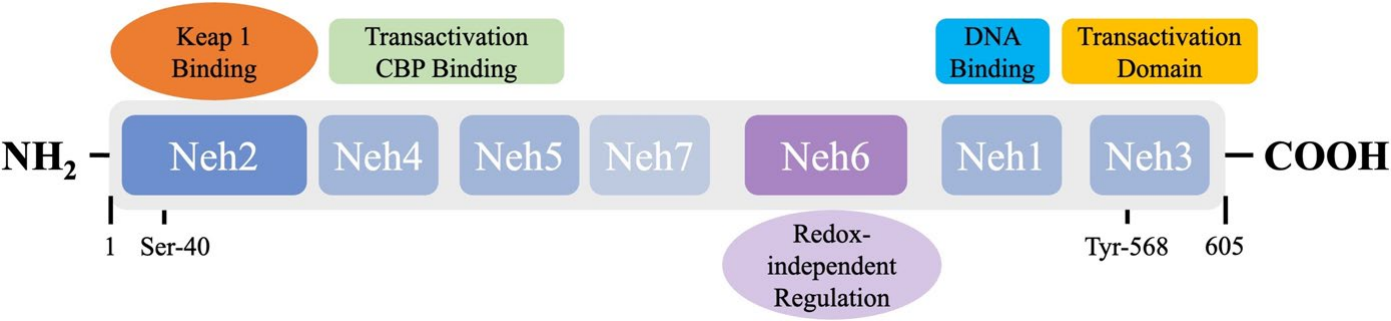
The structure and main functional areas of nuclear factor erythroid 2–related factor 2 (Nrf2) [64]

Nrf2, a member of the alkaline Leucine zipper family, is the most powerful anti-oxidative stress regulating transcription factor and is common in the body [58]. It consists of seven functional regions: Neh1–7. The Neh1 region contains a basic region-leucine zipper (bZIP). Once Nrf2 is activated, it enters the nucleus where bZIP interacts with Maf to form a heterodimer that prompts Nrf2 to recognize the (A/G) TGA DNA motif of antioxidant response elements (AREs) and bind to it to initiate transcription of the target gene [59]. The Neh1 region also contains a functional localization signal. The Neh2 region contains several lysine residues that can bind to ubiquitin to regulate Nrf2 degradation by protease. Neh2 is the binding site of Nrf2 and kelch-like ECH-associated protein 1 (Keap1) [60]. The Neh3 region is highly conserved, and its binding with the chromodomain helicase DNA binding protein 6 regulates the expression of Nrf2 target genes [61]. The Neh4 and Neh5 domains are associated with the initiation of downstream gene transcription. After Nrf2-Maf binds to the upstream ARE promoter, transcriptional activators such as cAMP responsive element binding protein bind to the Neh4 and Neh5 domains to initiate the transcription of downstream genes [62]. Neh6 is mainly involved in the degradation of Nrf2 under oxidative stress conditions (Fig. 2). When cells are attacked by oxidants, ser104 in Keap1 is mutated, leading to conformational changes in Keap1 that render it incapable of binding Nrf2, resulting in the release of Nrf2, which is then activated, enters the nucleus and binds Maf and ARE proteins, initiating the transcription of genes encoding antioxidant enzymes. It can improve the ability of cells and tissues to resist oxidative stress, thus exerting a protective effect in the body [63]. An abnormal Nrf2/ARE signaling pathway is associated with the occurrence and development of PD, and activation of the pathway can alleviate oxidative stress injury of cells and tissues [64].

## Mitochondrial dysfunction and oxidative stress

Mitochondria are major oxygen-consuming cells and contain oxidoreductases capable of transferring single electrons to oxygen molecules to generate ROS superoxide, including citric acid, pyruvate dehydrogenase, and glycerol-3-phosphate dehydrogenase [65]. The body is prone to generating ROS in the presence of electron carriers. Structurally and functionally intact mitochondria have antioxidant capacity sufficient to balance ROS production [66]. However, when they are damaged, ROS production and antioxidant capacity get off balance. ROS further damage mitochondria, producing more free radicals and weakening their antioxidant capacity, hence creating a vicious cycle [67, 68]. 1-methyl-4-phenyl-1,2,3,6-tetrahydropyridine (MPTP) is a neurotoxic contaminant of illicit opioids that crosses the blood-brain barrier [69]. Once oxidized to its active form, MPP+, it selectively damages DA neurons by blocking complex I in the electron transport system, depleting adenosine triphosphate (ATP) and increasing ROS within the organelle [70]. In addition, MPP + decreases the expression of mitochondrial genes and alters the levels of mitochondrial proteins. For example, exposure of differentiated SH-SY5Y cells to sublethal doses of MPP + severely impaired the overall respiratory properties of these cells, especially the function and stability of the inner mitochondrial membrane [71]. Furthermore, complex I activity in the brains of patients with PD is reduced by 30%, further confirming the link between complex I inhibition and the pathogenesis of PD [72].

## Dopaminergic neurons and oxidative stress

Substantia nigra pars compacta (SNpc) dopaminergic neurons have large, unmyelinated axons and higher energy requirements compared with other neurons [73]. SNpc is at a disadvantage in terms of energy balance, especially under oxidative stress, because energy generation and demand of SNpc can be easily unbalanced, resulting in energy demand exceeding supply. The negative energy balance further promotes oxidative stress and leads to the death of SNpc dopaminergic neurons. Therefore, the structure of DA neurons themselves determines their vulnerability to injury [74]. DA metabolism is also involved in oxidative stress [75]. In the resting state, DA is released by synaptic vesicle exocytosis and reabsorbed by cell membrane DA transporters, forming a DA transformation process. The loss of DA can be balanced by the synthesis of catecholamines in response to tyrosine dopamine [76]. Monoamine oxidase (MAO) is the mitochondrial enzyme responsible for the oxidative deamination of DA and is essential for DA metabolism [77]. Cytoplasmic DA produces dopa aldehyde (DOPAL) under the action of MAO, which is a strong oxidant that promotes the generation of ROS and causes oxidative damage to cells [78]. In addition, DOPAL is detoxified by acetaldehyde dehydrogenase to form 3,4-dihydroxyphenylacetic acid (DOPAC). When DA metabolism is impaired, DOPAC can undergo additional two-electron oxidation to generate ROS and dopaquinone, aggravating oxidative stress [79]. Moreover, in the substantia nigra glia, DA can also generate H2O2 with high permeability under the action of MAO, which cross-reacts with the surrounding dopaminergic cells to generate toxic hydroxyl radicals, leading to oxidative stress [80, 81].

## Calcium overload and oxidative stress

Mitochondrial oxidative stress in PD is closely associated with L-type Ca2 + channels, which differ from other neurons mainly in the the specificity of dopaminergic neurons [82]. SNpc dopaminergic neurons allow extracellular Ca2 + to enter the cytoplasm through L-type Ca2 + channels, maintaining sufficient DA synthesis levels. This process requires L-type Ca2 + channels to be open most of the time, leading to basal mitochondrial oxidative stress in SNpc dopaminergic neurons [83]. The overdependence of SNpc dopaminergic neurons on this Ca2 + channel is a key factor in Ca2+-mediated mitochondrial and endoplasmic reticulum stress [84]. In addition, overstimulation of ionotropic glutamate receptors can induce excess Ca2 + influx, causing oxidative stress-induced damage [85]. N-methyl-d-aspartate receptors (NMDARs) are major ionotropic glutamate receptors in the nervous system and are widely expressed in the basal ganglia, including the SNpc [86]. Under physiological conditions, NMDARs can lead to the production of normal ROS and mediate normal signaling pathways to support neuronal function and survival [87]. However, under neurodegenerative conditions, overactivation of extra-synaptic NMDARs leads to an influx of excess Ca2+, contributing to cell damage and death through oxidative stress [85]. Furthermore, Ca2 + requires stored ATP to enter neurons, and this reaction also leads to an increased burden on the ATP pump in the body, resulting in the production of more superoxide ions [88].

## Neuroinflammation and oxidative stress

Neuroinflammation causes neuronal loss in patients with PD [89]. Overactivated microglia release free radicals such as nitric oxide and superoxide, which induce and aggravate CNS oxidative stress [90]. In patients with PD, damaged dopaminergic neurons can release neuromelanin, α-synuclein, matrix metalloproteinase 3 (MMP-3), and other molecules that activate microglia [91, 92]. Neuromelanin includes dopamine oxide and protein and lipid peroxides and is one of the molecules responsible for chronic neuroinflammation in PD [93, 94]. Intracerebral injection of neuromelanin can induce intense activation of microglial cells in the substantia nigra (SN) and increase the release of nitric oxide [94]. α-synuclein can activate microglia and cause degeneration of dopaminergic neurons [95]. In addition, the isolated α-synuclein can also stimulate astrocytes to produce an inflammatory regulator, increasing activation, chemotaxis, and diffusion of microglia [96]. It is postulated that nitrifying α-synuclein may promote the occurrence of neuroinflammation by increasing the release of nitric oxide [97]. MMP-3 promotes the production of inflammatory factors in microglia [98]. In turn, cytokines produced by free radicals and inflammatory cells induce the production of MMP-3 [99]. Thus, there may be a cycle in which oxidative stress-induced MMP-3 activates microglia to produce free radicals and cytokines, and MMP-3 causes blood-brain barrier degradation and neutrophil infiltration, leading to neuroinflammation [56]. In dopaminergic neurons, MPP-3 also activates microglia to produce ROS [100].

## Gene mutations and oxidative stress

Human mtDNA is compact, has no introns, lacks histone protection and repair systems, and is adjacent to the respiratory chain, making it vulnerable to oxidative stress [101]. ROS produced by the mitochondrial respiratory chain is the most common cause of mtDNA mutations [102]. ROS induce cell membrane damage, which increases the mutation rate of mtDNA in tissues [103]. Due to the high content of easily oxidized unsaturated fatty acids and low antioxidant enzymes in the CNS, neurons in the CNS are more vulnerable to oxidative stress-induced damage, and their mtDNA mutation rate is also higher than that in ordinary tissues [104]. The mtDNA mutation rate has been correlated with the incidence of PD [105].

In patients with PD, the genes *DJ-1*, *parkin*, and *PINK1* are associated with mitochondrial function [106]. *DJ-1* prevents cell death due to oxidative stress and can be used as a redox component [107]. Mice with *DJ-1* mutations have increased oxidative DA production and significantly increased mitochondrial oxidative stress, suggesting that the decline in *DJ-1* function can promote PD resulting from oxidative stress [108]. Uncoupling proteins (UCP) are ion channels in the mitochondria whose opening rate increases with an increase in peroxides [109]. ROS production increased in the mitochondria of *DJ-1*-deficient SNpc dopaminergic neurons, indicating reduced UCP defense function [110]. *PINK1* and *Parkin* can regulate mitochondrial quality and detect mitochondrial dysfunction. *Parkin* and *PINK1* knockout or mutant mice showed mitochondrial damage in the CNS [111].

## Metal ion concentrations and oxidative stress

The CNS is the main storage site for iron, copper, zinc, manganese, and other metal ions that are involved in various physiological activities in the nervous system [112]. Iron is involved in oxygen transport and storage, mitochondrial respiration, and DNA synthesis [113]. Excess iron stimulates Fenton reaction and produces excess ROS, which causes strong CNS oxidative stress and leads to neuron cell damage [114]. In addition, excess iron also reduces the ratio of reduced glutathione to oxidized glutathione, damages the glutathione-dependent antioxidant defense system, induces lipid peroxidation, and ultimately leads to the degeneration and death of dopaminergic neurons [115]. Copper ions are mainly distributed in the locus coeruleus and substantia nigra in the brain [116]. Copper ions have redox potential and can be used as cofactors or structural components of various enzymes, and are involved in cellular respiration, free radical detoxification, iron metabolism, and neurotransmitter synthesis [117]. Oxidative damage to dopaminergic neurons can be caused by increased levels of free copper ions in the CNS [118]. Copper ions inhibit glutathione peroxidase (GSH-1) expression in dopaminergic neurons, changing the structure of dopaminergic neurons in basal ganglia and leading to PD [119]. In redox reactions containing reducing agents such as superoxide, glutathione, or ascorbic acid, copper can catalyze H2O2 to generate hydroxyl radicals through the Haber-Weiss cycle and induce DNA oxidation and breakage, resulting in oxidative damage to dopaminergic neurons [120]. Manganese is an important micronutrient that is mainly distributed in the globus pallidus and caudate nucleus in the brain [121]. Manganese ions are commonly taken up by dopaminergic neurons and can accumulate in the mitochondria or nucleus via divalent cation transporters [122]. Manganese neurotoxicity may result from its affinity for regions with high levels of neuromelanin and its tolerance to multiple oxidation environments, leading to the auto-oxidation of DA and the production of ROS [123]. Manganese often destroys the synaptic function of dopaminergic neurons by promoting oxidative stress responses and affecting mitochondrial function [124].

**Fig. 3:**
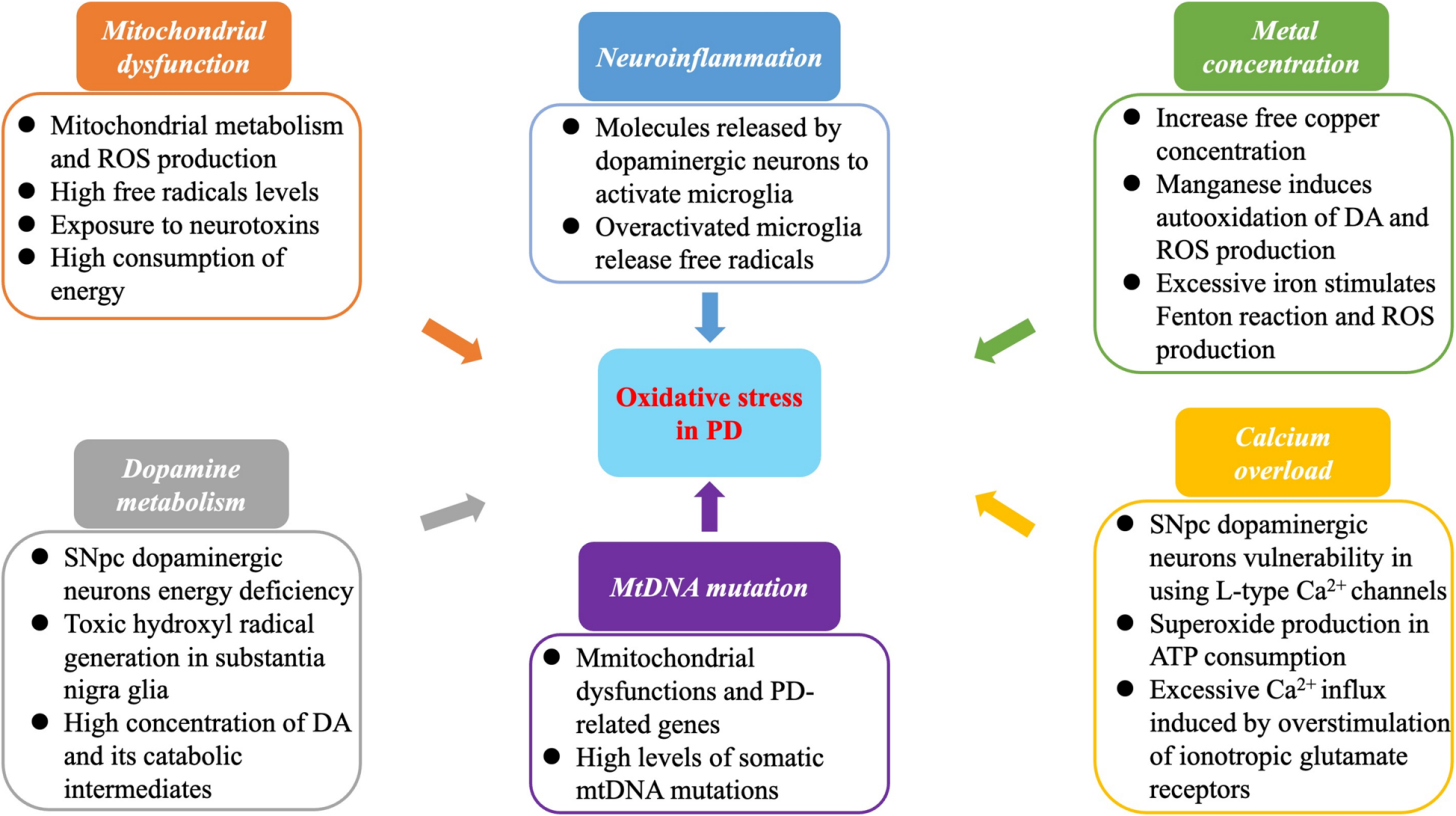
Major causes of oxidative stress in Parkinson’s disease (marked with different colors). Each group describes general biological processes that may lead to excessive oxidative stress, contributing to the pathogenesis of Parkinson’s disease. ATP: adenosine triphosphate; DA: dopamine; mtDNA: mitochondrial DNA; PD: Parkinson’s disease; ROS: reactive oxygen species; SNpc: Substantia nigra pars compacta

The pathogenesis of PD is closely associated with oxidative stress caused by mitochondrial dysfunction, dopamine metabolism, neuroinflammation, mtDNA mutation, and abnormal metal ion concentrations (Fig. 3). There is currently no effective method for delaying the neurodegenerative process of PD. Some antioxidants are thought to have neuroprotective effects but do not have the desired effect of stopping PD progression. Therefore, regulating oxidative stress at the transcriptional/post-transcriptional level may be a new approach to treating PD.

## Interplay between LncRNAs and oxidative stress in PD

**Fig. 4:**
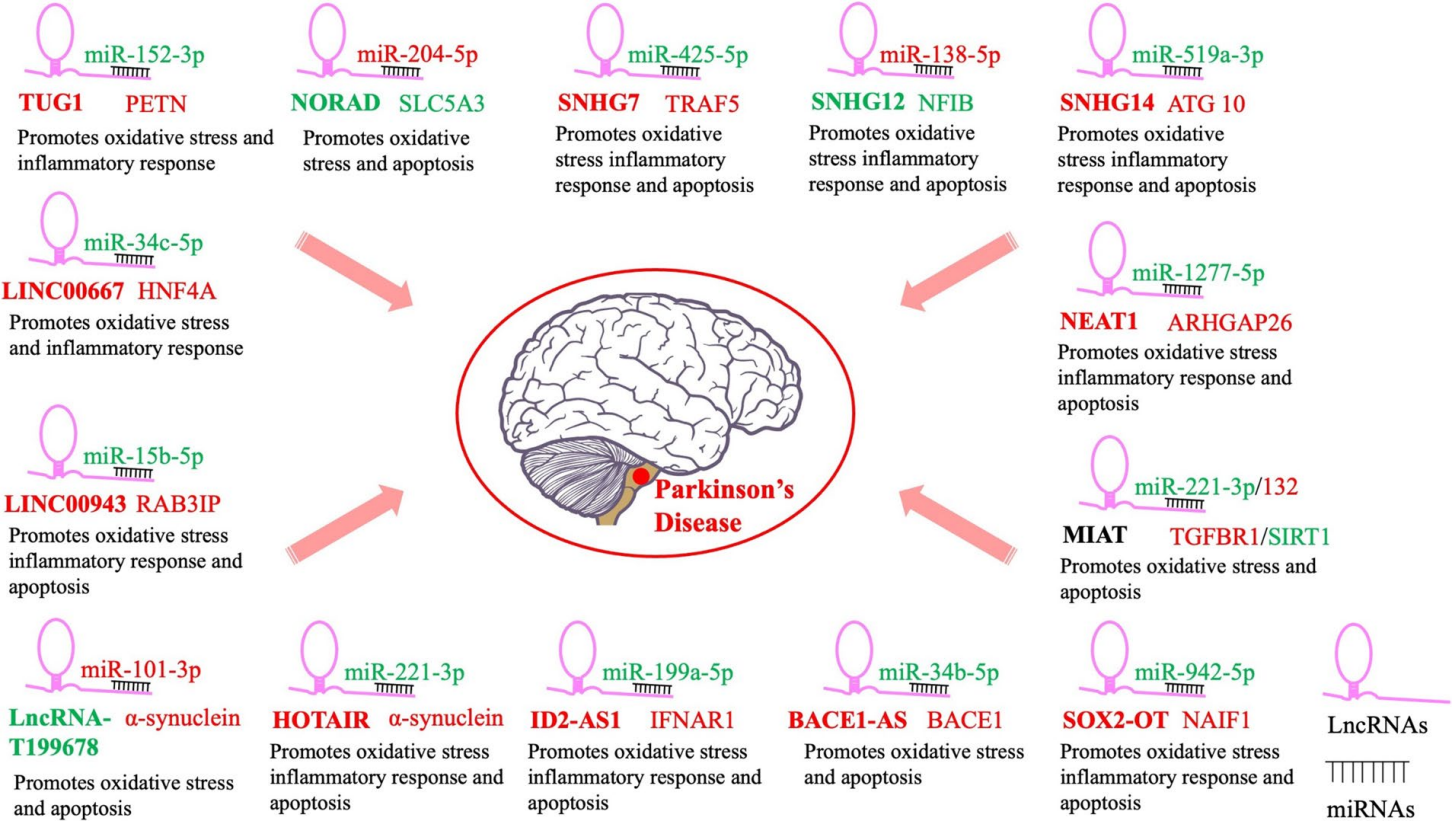
LncRNAs cause oxidative stress in PD through competitive endogenous RNA regulation of the expression of miRNAs and their target genes. Red and green colors represent high and low expression, respectively. LncRNAs, long noncoding RNA; PD: Parkinson’s disease

Emerging studies suggest that regulating the expression of some lncRNAs may modulate the oxidative stress response in the CNS, relieve pathological oxidative stress injury, and exert neuroprotective effects [125, 126]. However, confirming the role of lncRNAs in oxidative stress response is not easy and further research on the overall regulatory mechanism of lncRNAs is required. Below, we review the current literature on the interaction between oxidative stress and lncRNAs in PD (Fig. 4).

MPP+-induced models showed severe oxidative stress and increased inflammatory response [127]. Zhai et al. suggested that expression of the lncRNA taurine upregulated gene 1 (TUG1) was upregulated in MPP+-induced SH-SY5Y cells and an MPTP-stimulated PD mouse model. Downregulated TUG1 expression suppressed MPP+-induced cytotoxicity, and this was supported by increased cell viability and reduced ROS. Moreover, TUG1 downregulation alleviated neuroinflammation of MPP+-induced cells by reducing TNF-α and IL-1β expression. The expression of miR-152-3p, which acts as a TUG1 sponge, decreased and modulated the pathological damage in the substantia nigra of PD mice in vivo. These results provide a novel understanding of the effect of TUG1 on PD progression [128] (Table 1). The lncRNA activated by DNA damage (NORAD) is also involved in regulating MPP+-induced cytotoxicity in an in vitro model of PD. Upregulated NORAD expression alleviated MPP+-induced apoptosis and mitochondrial dysfunction, as evidenced by decreased ROS activity, lactate dehydrogenase (LDH) levels, and caspase 3/7 activities, indicating that NORAD can regulate intrinsic onset, development, or inhibition of PD-associated mitochondrial or apoptotic signaling pathways [129]. Zhou et al. obtained similar results, showing that NORAD overexpression alleviated MPP+-induced oxidative stress, cytotoxicity, and inflammatory responses in SK-N-SH/SK-N-AS cells. Furthermore, NORAD serves as a specific sponge for miR-204-5p and can act as a moderator, attenuating MPP+-induced oxidative stress and reducing ROS production, superoxide dismutase (SOD), and cytotoxic events, including apoptotic motivation, cell viability inhibition, and LDH release [130].

**Table 1:** LncRNAs associated with Parkinson’s disease and implicated in regulation of oxidative stress and related cellular pathways

lncRNAs	Expression	Animals or cells models	Downstream targets	Anti-PD mechanism and observed indicators	References
TUG1	Increased	SH-SY5Y cells/ C57BL/6J mice	miR-152-3p/PETN	Inhibits MPP^+^/MPTP-induced oxidative stress and decreases inflammatory response, reduces ROS levels.	Zhai et al. [128]
NORAD	Decreased	SH-SY5Y cells	NA	Inhibits MPP^+^ induced cytotoxicity, reduces ROS activities and LDH levels.	Song et al. [129]
NORAD	Decreased	SK-N-SH/SK-N-AS cells	miR-204-5p/SLC5A3	Attenuates MPP+-induced oxidative stress (reduced ROS, production of SOD) and cytotoxic events (including apoptotic motivation, inhibition of cell viability, and LDH release).	Zhou et al. [108, 130]
SNHG7	Increased	SH-SY5Y cells/ SD rats	miR-425-5p/TRAF5	Reduces LDH expression, attenuates MDA level, enhances SOD and GSH-PX level. Attenuates inflammatory responses, oxidative stress, and increases cell viability, inhibits cell apoptosis.	Zhang et al. 2020
SNHG12	Decreased	SH-SY5Y cells	miR-138-5p/NFIB	Increases cell viability, LDH and SOD activity, decreases ROS level, cleaved-caspase3/caspase3 ratio, and inhibits inflammatory response and cell apoptosis.	Yan et al. [131]
SNHG14	Increased	SH-SY5Y cells	miR-519a-3p/ATG10	Attenuates MPP+-induced cell damage by modulating cell cycle arrest, cell viability, oxidative stress, and apoptosis. Reduces LDH activity and ROS generation, and enhances SOD activity.	Zhuang et al. [133]
NEAT1	Increased	SK-N-SH cells	miR-1277-5p/ARHGAP26	Increases cell viability, Bcl-2 expression, LDH, SOD and GSH-PX levels, decreases Bax, cleaved caspase-3, IL-6, IL-1β, TNF-α, and MDA. Inhibits apoptosis, inflammatory response, and oxidative stress.	Zhou et al. [136]
MIAT	Increased	SH-SY5Y cells/ C57BL/6J mice	miR-221-3p/TGFBR1	Inhibits MPP+– induced oxidative stress and apoptosis in neurons, decreases the expression of cleaved caspase-3 and Bax/Bcl-2, reduces LDH activity and ROS generation, promotes the production of SOD and GSH, inhibits MDA levels, suppresses TGF-β1 but increased Nrf2 expression.	Lang et al. [137]
MIAT	Decreased	PC12 cells	miR-132/SIRT1	Inhibits MPP+– induced oxidative stress in PC12 cells. Increases the SOD activity, GSH-PX level, and the Bcl-2/Bax ratio, decreases cleaved-caspase 3 expression, increases cell viability, inhibits cell apoptosis and oxidative stress.	Xu et al. [138, 145]
SOX2-OT	Increased	SH-SY5Y cells	miR-942-5p/NAIF1	Decreases LDH activity, cleaved caspase-3 expression, reduces the levels of TNF-α, IL-1β, ROS and increases SOD activity. Inhibit oxidative stress, inflammation, and neuronal apoptosis.	Guo et al. [140]
BACE1-AS	Increased	SD rats	miR-34b-5p/BACE1	Reduces the levels of MDA and iNOS, increase the levels of GSH-PX, SOD and DA inhibits the apoptosis of SN neurons in PD rats and improves oxidative stress injury.	Li et al. [141]
UCA1	Increased	Wistar rats	PI3K/Akt signaling pathway	Inhibits cleaved-caspase 3, Bax expression, promotes Bcl-2 expression, up-regulates BDNF and NGF expression, increases the activities of SOD and GSH-PX, decreases the content of MDA, and TNF-α, IL-6 and IL-1β levels. Reduces apoptosis and oxidative stress, and improves neuroinflammation.	Cai et al. [143]
ID2-AS1	Increased	SH-SY5Y cells	miR-199a-5p/IFNAR1	Elevates Bcl-2 and reduces Bax expression, and ROS, TNF-α, IL-6, IFN-γ levels, inhibits apoptosis, reduces the inflammation, and attenuates the oxidative stress on PD cell.	Xu et al. [138, 145]
HOTAIR	Increased	SH-SY5Y cells	miR-221-3p/α-synuclein	Decreases the cleaved-casp-3, Bax protein levels, LDH, ROS, and increases the Bcl-2 protein and SOD level. Attenuates the oxidative stress and inhibits apoptosis in MPP + -treated SH-SY5Y cells.	Sun et al. [147]
LncRNA-T199678	Decreased	SH-SY5Y cells	miR-101-3p/α-synuclein	Increases level of ROS, reverses the oxidative stress, cell cycle abnormality, and apoptosis induced by α-synuclein	Bu et al. [148]
LINC00943	Increased	SH-SY5Y cells	miR-15b-5p/RAB3IP	Reduces Bax, cleaved-caspase 3, LDH and MDA levels, inhibits TNF-α, IL-1β, IL-6 levels, increases Bcl-2 expression, and SOD and GSH-PX levels. Inhibits apoptosis, inflammatory injury and oxidative stress of MPP + treated SH-SY5Y cells.	Meng et al. [149]
LINC00667	Increased	SH-SY5Y cells	miR-34c-5p/HNF4A	Depletes on ROS generation and promoting effect on SOD activity, declines TNF-a, IL-1b, and IL-6. Inhibits inflammatory injury, oxidative stress of MPP + treated SH-SY5Y cells.	Huo et al. [150]

Akt: protein kinase B; BDNF: brain-derived neurotrophic factor; GSH-PX: glutathione peroxidase; IL: interleukin; LDH: lactate dehydrogenase; MDA: malondialdehyde; MPP^+^: 1-Methyl-4-phenylpyridinium ion; MPTP: 1-methyl-4-phenyl-1,2,3,6-tetrahydropyridine; NGF: nerve growth factor; NOS3: nitric oxide synthase 3; Nrf2: nuclear factor E2-related factor 2; PD: Parkinson’s disease; PI3K: phosphatidylinositol-4,5-bisphosphate 3-kinase; ROS: reactive oxygen species; SOD: superoxide dismutase; tBHP: tert-butyl hydroperoxide; TGF-β1: transforming growth factor-β1; TNF-α: tumor necrosis factor-α

Expression of the lncRNA small nucleolar RNA host gene 7 (SNHG 7) was upregulated in patients with PD. Downregulating SNHG7 in a PD rat model reduced LDH expression, attenuated malondialdehyde (MDA) levels, and enhanced SOD and glutathione peroxidase (GSH-PX) levels. SNHG7 inhibition in a PD cell model attenuated inflammatory responses and oxidative stress, increased cell viability, and inhibited cell apoptosis. These results suggest that regulating SNHG7 expression may have a protective role against PD through various mechanisms. Further exploration of the regulatory mechanism underlying SNHG7 showed that SNHG7 promoted inflammation and oxidative stress mediated by TRAF5 through sponging of miR-425-5p as a competitive endogenous RNA (ceRNA). SNHG7 inhibition improved PD neuronal apoptosis by alleviating the regulation of inflammation and oxidative stress via the miR-425-5p/TRAF5/NF-κB signaling pathway [131]. MPP + treatment of SH-SY5Y cells reduced SNHG12 expression and enhanced miR-138-5p expression in a PD cell model. Yan et al. observed that upregulated SNHG12 expression increased cell viability and LDH and SOD activities, but decreased ROS levels and the cleaved-Caspase3/Caspase3 ratio and inhibited TNF-α and IL-1β inflammatory responses, as well as apoptosis in MPP+-stimulated SH-SY5Y cells. miR-138-5p was identified as the SNHG12 target, and SNHG12 was shown to exert a neuroprotective effect by sponging miR-138-5p, although the exact mechanism needs to be studied further [132]. SNHG14 expression was dramatically enhanced in the same PD cell model. Moreover, knocking down SNHG14 attenuated MPP+-induced SK-N-SH cell damage by modulating cell cycle arrest, cell viability, oxidative stress, and apoptosis. SNHG14 and ATG10 are ceRNA of miR-519a-3p, and SNHG14 can positively regulate the expression of ATG10 by sponging miR-519a-3p. Zhuang et al. pointed out that targeted silencing of SNHG14 and restoration of miR-519a-3p prevented MPP+-induced toxicity against dopaminergic neurons by regulating ATG10 [133].

Nuclear enriched assembly transcript 1 (NEAT1) has recently been implicated in the regulation of mitochondrial formation and function [134]. NEAT1 expression increased under exposure to oxidation inducers [135]. NEAT1 knockdown may play a protective role in PD through transcription and post-transcription or by influencing the transcriptome. For example, NEAT1 antagonized MPP+-induced inflammatory responses, oxidative stress, and apoptosis in SK-N-SH cells by inhibiting the expression of miR-1277-5p [136].

The lncRNA myocardial infarction-associated transcript (MIAT) was highly expressed and promoted neuronal inflammation and oxidative stress in PD SH-SY5Y cells and a C57BL/6J mouse model. miR-221-3p was identified as a target of MIAT, and inhibiting MIAT suppressed TGFBR1 expression and TGF-β1 but increased Nrf2 expression by sponging miR-221-3p [137]. However, a study by Xu et al. reached a contrary conclusion. They observed that MIAT expression significantly decreased in MPP+-induced PC12 cells, and upregulation of MIAT expression inhibited MPP+-induced oxidative stress and exerted a cytoprotective effect. Further exploration of the cytoprotective mechanism underlying MIAT found that MIAT may exert a cytoprotective effect by competitively inhibiting the expression of miR-132 and upregulating SIRT1, the target gene of miR-132 [138].

SRY-box transcription factor 2 overlapping transcript (SOX2-OT) is strongly associated with poor clinical outcomes in cancer patients, and its role as an oncogene and cancer prognostic or diagnostic biomarker has been established [139]. However, the role of SOX2-OT in PD pathogenesis remains unclear. MPP + treatment upregulated SOX2-OT expression, increased apoptosis, and reduced the viability of SH-SY5Y cells. SOX2-OT downregulation significantly decreased LDH activity and cleaved caspase-3 expression, reduced the levels of TNF-α, IL-1β and ROS, and increased SOD activity in SH-SY5Y cells. Guo et al. showed that SOX2-OT is involved in the occurrence and development of PD by directly regulating the miR-942-5p/NAIF1 signaling axis, inhibiting oxidative stress, inflammation, and neuronal apoptosis [140].

The mechanism underlying the action of the lncRNA β-Site amyloid precursor protein cleaving enzyme 1 antisense transcript (BACE1-AS) on PD progression is currently unclear. Li et al. demonstrated that BACE1-AS is overexpressed in PD, and BACE1-AS downregulation reduced MDA and inducible nitric oxide synthase levels; increased GSH-PX, SOD, and DA levels; inhibited the apoptosis of SN neurons in PD rats; and improved oxidative stress injury. They also showed that BACE1-AS specifically bound to and was a direct target of miR-34b-5p. Silencing BACE1-AS ameliorated oxidative stress injury in PD rats by upregulating miR-34b-5p and downregulating BACE1 [141].

Urothelial carcinoma-associated 1 (UCA1) is an oncogene that promotes tumor cell proliferation, migration, and invasion while inhibiting tumor cell apoptosis [142]. UCA1 expression was upregulated in the SN of rats with PD and was involved in the occurrence and development of PD. UCA1 downregulation can improve neurobehavioral changes in rats with PD. In addition, UCA1 silencing reduced SN neuron apoptosis and oxidative stress, and improved neuroinflammation in rats with PD by inhibiting the activation of the phosphatidylinositol-4,5-bisphosphate 3-kinase (PI3K)/protein kinase B (Akt) signaling pathway [143].

Few studies have analyzed the role of the lncRNA ID2-AS1 in human diseases. However, one study showed that ID2-AS1 expression was downregulated in metastatic liver cancer tissues and cell lines, indicating that ID2-AS1 may act as a tumor suppressor, inhibiting the invasion and metastasis of hepatocellular carcinoma cell lines [144]. ID2-AS1 expression was significantly upregulated in MPP+-induced cells in a concentration- and time-dependent manner. ID2‑AS1 suppression attenuated the neuronal damage caused by exposure to MPP+, including reduced cell viability, increased cell death and apoptosis, increased production of inflammatory cytokines, and increased oxidative stress. Mechanically, downregulated ID2-AS1 played a protective role in the miR-199a-5p/IFNAR1 axis by regulating the JAK2/STAT1 signaling pathway [145].

HOX transcript antisense RNA (HOTAIR) is an lncRNA that is upregulated in PD and can regulate the progression of PD [146]. However, the specific role of HOTAIR in PD and its underlying molecular mechanism remain unclear. Knocking down HOTAIR in MPP+-induced SH-SY5Y cells increased cell viability, decreased cell apoptosis, and reduced the secretion of inflammatory factors and oxidative stress response. In addition, HOTAIR sponged miR-221-3p, directly targeting α-synuclein and regulating its expression. Therefore, HOTAIR can alleviate MPP+-induced SH-SY5Y cell damage through the miR-221-3p/α-synuclein axis, suggesting that HOTAIR has potential value in PD treatment [147].

Other lncRNAs that are still being studied may also affect the occurrence and development of PD by influencing oxidative stress. For example, lncRNA-T199678 alleviated α-synuclein-induced dopaminergic neuron damage by targeting miR-101-3p, promoting PD [148]. Silencing LINC00943/LINC00667 alleviated MPP+-induced neuronal injury, inflammatory responses, and oxidative stress and decreased cell viability [149, 150].

This review clearly illustrates the relationship between oxidative stress and lncRNAs in PD. However, many experimental details and technical issues need to be resolved before lncRNAs-based therapies can be applied in the clinic. First, most studies on the relationship between lncRNAs and oxidative stress have used cell cultures. Caution should be exercised when translating findings obtained from cell cultures to human neurons. Second, despite improvements in computational algorithms, the prediction and validation of lncRNA-miRNA-mRNA regulation remain challenging [151]. Third, further studies are needed to improve our understanding of the underlying molecular mechanisms through which oxidative stress, mitochondrial dysfunction, and lncRNAs are linked to specific pathologies.

## Conclusions

Oxidative stress plays a critical role in the occurrence and development of PD. Furthermore, secondary pathological damage accompanied by oxidative stress, including neuroinflammation, mitochondrial damage, and increased apoptosis, is an important mechanism that promotes PD progression. Therefore, alleviating oxidative stress in PD may help alleviate neuronal damage, making it a novel therapeutic strategy for PD. Recent studies have shown that lncRNAs are widely involved in several human diseases, including PD, and are associated with oxidative stress. Our review showed that many lncRNAs regulate oxidative stress by interacting with miRNAs to promote PD progression. Additionally, some lncRNAs can directly regulate mitochondrial function and integrity, thereby participating in the pathological mechanism of PD by modulating energy metabolism and promoting ROS generation. These oxidative stress-related lncRNAs are potential key biomarkers and therapeutic targets for PD.

## Data Availability

The datasets used and/or analyzed during the current study are available from the corresponding author on reasonable request.

## Abbreviations

PD: Parkinson’s disease
lncRNAs: Long noncoding RNAs
DA: Dopamine
ROS: Reactive oxygen species
CNS: Central nervous system
mtDNA: Mitochondrial DNA
MPP-3: Matrix metalloproteinase 3
bZIP: Basic region-leucine zipper
AREs: Antioxidant response elements
Keap1: Kelch-like ECH-associated protein 1
MPTP: 1-methyl-4-phenyl-1,2,3,6 tetrahydropyridine
ATP: Adenosine triphosphate
MAO: Monoamine oxidase
DOPAL: DA produces dopa aldehyde
DOPAC: 3,4-dihydroxyphenylacetic acid
NMDARs: N-methyl-d-aspartate receptors
MMP-3: Metalloproteinase 3
SN: Substantia nigra
UCP: Uncoupling proteins
GSH-1: Glutathione peroxidase
TUG1: Taurine upregulated gene 1
LDH: Lactate dehydrogenase
SNHG 7: Small nucleolar RNA host gene 7
GSH-PX: Glutathione peroxidase
ceRNA: Competitive endogenous RNA
NEAT1: Nuclear enriched assembly transcript 1
MIAT: Myocardial infarction-associated transcript
SOX2-OT: SRY-box transcription factor 2 overlapping transcript
BACE1-AS: β-Site amyloid precursor protein cleaving enzyme 1 antisense transcript
UCA1: Urothelial carcinoma-associated 1
PI3K: Phosphatidylinositol-4,5-bisphosphate 3-kinase
Akt: Protein kinase B
HOTAIR: HOX transcript antisense RNA
